# Application of implementation science frameworks to a community-based healthy eating and activity intervention: a cross-sectional analysis

**DOI:** 10.3389/frhs.2026.1637060

**Published:** 2026-02-18

**Authors:** Rachel G. Tabak, Cynthia D. Schwarz, Debra Haire-Joshu, Jinli Wang, Amanda Gilbert, Karen Steger-May

**Affiliations:** 1 School of Public Health, Washington University in St. Louis, St. Louis, MO, United States; 2 Washington University in St Louis School of Medicine, St. Louis, MO, United States

**Keywords:** Consolidated Framework for Implementation Research (CFIR), Reach, Effectiveness, Adoption, Implementation, Maintenance (RE-AIM) implementation science frameworks, community setting and healthy eating and activity

## Abstract

**Introduction:**

To contribute to the growing literature applying implementation science frameworks, this study utilized the Consolidated Framework for Implementation Research (CFIR) and the Reach, Effectiveness, Adoption, Implementation, and Maintenance (RE-AIM) frameworks. Our objectives were to (1) describe surveys used to assess CFIR context and RE-AIM implementation outcomes and (2) explore correlations between constructs within and across the frameworks in a community-based home-visiting organization.

**Methods:**

This cross-sectional, baseline secondary analysis was conducted within a cluster-randomized trial partnered with parents as teachers (PAT), a national home-visiting, community-based organization. Guided by CFIR and RE-AIM, parent educators (provider level: seven scales, 26 items) and one leader per site (organization level: 11 scales, 56 items) completed surveys online before beginning the study. Standardized Cronbach coefficient alphas were calculated to reflect internal consistency reliability and intraclass correlation coefficients (ICCs) were used to reflect the agreement among parent educators within sites. Relationships between contextual factors (CFIR), antecedent outcomes (CFIR), and RE-AIM outcomes were calculated using Pearson correlations.

**Results:**

A total of 271 parent educators and 26 site leaders completed the demographic survey. Most parent educators (72.5%) were white and a third of parent educators identified as Hispanic or Latino. Alphas ranged from 0.70 to 0.92 and 0.47 to 0.95 for scales completed by parent educators and site leaders, respectively. There was limited agreement among parent educators within sites; ICCs ranged from 0 to 0.24. Correlations for constructs within CFIR and between CFIR context and CFIR antecedent outcomes were statistically significant, while few correlations with these constructs and those in RE-AIM were statistically significant.

**Discussion:**

This study demonstrates the application of CFIR, CFIR outcomes addendum, and RE-AIM. Investigators can use these findings to inform future studies incorporating implementation science in nutrition and physical activity research and to examine theoretical relationships between constructs and frameworks.

## Introduction

People experiencing the greatest social determinants of health-related barriers are often less connected to healthcare, meaning that access to healthy eating and physical activity interventions in clinical settings may not reach those who can benefit most (1–7). Extending health promotion interventions beyond healthcare settings is therefore critical to prevent chronic disease, such as cardiovascular disease and diabetes, at a population level (8, 9). Community settings (i.e., organizations outside clinical healthcare or public health agencies that influence the everyday lives of individuals) are ideal places to reach families with young children, meeting them where they live, work, learn, and play (10, 11). Given the reach/access of community-based interventions, it is important to ensure that their delivery is optimized. Implementation science provides methods and theories that assess contextual factors that are important for implementation (12–25). Implementation science emphasizes organizational and provider-level contextual factors, which increase the relevance of interventions embedded within organizations.

Frameworks from implementation science provide constructs that are important in understanding the context in which an intervention is implemented as well as the evaluation of implementation efforts (26–28). A commonly cited (27, 29–32) framework from implementation science is the Consolidated Framework for Implementation Research (CFIR) (32–37). CFIR has been increasingly applied in community-based health promotion (18, 24, 38–40) to understand context (37) (e.g., leadership engagement, available resources, mission alignment), as well as antecedent outcomes (potential predictors of adoption or implementation), including the implementation climate (32–37). This framework is frequently combined with the RE-AIM (Reach, Effectiveness, Adoption, Implementation, and Maintenance) framework (18, 32, 41–50), which supports evaluation of implementation outcomes (e.g., adoption) (42, 51–55), particularly in healthy eating and physical activity promotion research (24, 56). An important way to operationalize frameworks and move toward theorizing is through the measurement of their constructs and exploring potential relationships (26, 52, 57–60). Since many constructs central to implementation science frameworks involve the perceptions of individuals (e.g., providers) engaged in implementation, there have been calls to report on the use of survey-based assessments (58).

Most existing implementation science measures have been applied in clinical settings, which differ in important ways from community settings. Differences between clinical and community settings include funding streams, types of staff, and physical spaces (9). Even outside of clinic care, most interventions in community settings have been studied in schools. For example, a review by Spiga et al. found that of 172 studies reviewed, only 15 were conducted in community settings and eight were conducted at home, whereas 111 studies were conducted in schools (61). Other organizations outside of clinical settings (e.g., home visiting organizations, social service agencies, and housing authorities) differ in important ways from schools in their heterogeneous structures and staffing. This leaves a gap in measurement in community organizations. To rigorously assess implementation context and outcomes, researchers have developed survey-based quantitative measures, which can be used in community-based healthy eating and activity promotion studies (62). Empirical use of such measures is needed to build an understanding of the internal consistency of the measures and the extent to which community-providers agree on measures of implementation context and outcomes within their community-based sites (15, 16, 63). Furthermore, applying frameworks by measuring constructs and testing relationships in community-based studies is essential to developing a generalizable understanding of implementation outside the clinical setting (28, 56, 64).

This study presents a secondary analysis of baseline data from an ongoing cluster-randomized trial testing the Healthy Eating and Active Living Taught at Home (HEALTH) (blinded trial registration) intervention across home-visiting sites nationwide. Specifically, this study uses baseline data from a nationwide home-visiting trial to (1) describe survey measures of CFIR and RE-AIM constructs (including reliability and within-site agreement) and (2) explore correlations between these constructs to advance understanding of implementation in community-based organizations.

## Materials and methods

### Design and setting of the study

This is a cross-sectional, secondary analysis of baseline data from a pragmatic cluster-randomized controlled trial testing the HEALTH intervention, conducted between 2018 and 2025. The trial was conducted in partnership with parents as teachers (PAT) (65), a national home-visiting, community-based organization. HEALTH is an evidence-based intervention (66) that embeds healthy eating and active living content into the PAT model. PAT is delivered through sites in all 50 U.S. states, located in a variety of settings outside of clinical care, such as social service organizations and educational settings (e.g., early childhood organizations). The providers delivering PAT are parent educators (PEs), who are trained to deliver the PAT curriculum, reside in the communities they serve, and maintain relationships with families over time. A total of 43 PAT sites across the country participated in this study. Randomization to the HEALTH training curriculum—to support implementation of the HEALTH intervention or to usual care PAT—occurred at the site level. PEs at sites randomized to the HEALTH arm received training in delivering the HEALTH curriculum to the families they serve; PEs at sites randomized to usual care continued to deliver PAT as usual. The trial considered three levels: mother, parent educator (provider), and PAT site (organization). This analysis focused on two levels of analysis: PEs (assessed through a PE survey) and PAT sites (assessed through a site leader survey).

### Participants and procedures

PEs and site leaders completed a consent process with study team members by phone. Survey measures were completed by PEs (to collect provider level) and site leaders (to collect data about their site, representing the organization level) via online surveys in English using REDCap (67). Respondents received $15 remuneration for completing the survey. For the current analysis, we combined the survey respondents for the HEALTH and Usual Care groups since these data were collected prior to detailed study-related training. The study protocol was reviewed and approved by the University Human Research Protection Office (#201810157).

### Measures

The overall study was guided by the CFIR (to understand contextual factors that impact implementation and implementation outcomes) and RE-AIM (implementation outcomes). These frameworks were used to guide the selection of measures throughout the development, conduct, and analysis of the trial. Existing survey measures were identified for relevant constructs for each framework, based on the literature and the perspectives of PEs and site leaders (42, 68–70). These surveys were initially employed and refined in a small study incorporating qualitative and quantitative methods with PEs (*n* = 6) and site leaders (*n* = 10) (68). Items refer to both current PAT practices (e.g., Inner setting culture: Our site encourages everyone to share ideas) and prospective HEALTH adoption (e.g., Adoption: It was intuitively appealing). A complete list of the scales used to measure each construct, the individual questions, and the response options for each scale are described in Supplementary Table 1.

### Statistical methods

Scale scores were calculated as the mean of the items in the scale, requiring non-missing data for each item in the scale. Means with standard deviations (SD) or medians with interquartile range and ranges were reported for each scale. Standardized Cronbach’s coefficient alpha is a measure of reliability, representing the internal consistency of the items within the scale (71). We categorized the internal consistency reliability as follows: Poor Cronbach's alpha < 0.50; Minimal/Emerging Cronbach's alpha values =0.50–0.69; Adequate Cronbach's alpha values of =0.70–0.79; Good Cronbach's alpha values of =0.80–0.89; and Excellent Cronbach's alpha values of ≥0.90 (72).

Intraclass correlation coefficients (ICCs) (73) were calculated to reflect the agreement of PEs within a site and were calculated for the derived scale scores. Sites with fewer than three PE respondents were excluded from the ICC analyses. ICC (1,1) for single measurements was estimated using a one-way analysis of variance with a random effect for respondents. We selected this type of ICC because the unit of analysis was the response from each PE (not of a mean across PEs), and a different set of PEs responded for each site, who we considered randomly selected from a larger population of PEs for the site. ICCs reflected the reliability of the individual PE responses and required data from the same number of PEs for each site. As such, we randomly selected two PEs from each site for 30 iterations, so that ICCs were reported for 30 replicate pairings of two PEs. PEs with missing scale scores were excluded. There is no rule to define “acceptable” agreement, as this depends upon the use of the measurements and how much agreement is expected from PEs within a site (74); however, the following standards were applied: virtually none to slight reliability ≤0.40; fair reliability = 0.41–0.60; moderate reliability = 0.61–0.80; and substantial reliability = 0.81–1.0.

Supplementary Figure 1 (Supplementary File 2) illustrates the conceptual framework integrating CFIR context constructs, CFIR antecedent assessments, and RE-AIM implementation outcomes to guide the relational analysis. Correlations between contextual factors (CFIR), antecedent outcomes (CFIR), and RE-AIM outcomes were analyzed using Pearson correlations. Bivariate plots were examined to assess linearity. Correlations were considered significant if the *p*-value was less than.05. All data analyses were conducted using SAS software, version 9.4 for Windows (SAS Institute Inc., Cary, NC, USA).

## Results

Of the 43 sites enrolled in the study, 39 sites had baseline PE and site leader data (Table 1). Across both groups, 284 PEs consented to participate, 271 PEs responded to the baseline survey, and 269 PEs completed the demographic survey form. Among site leaders, 30 consented to participate, and 26 responded to the baseline survey and completed the demographic survey. On average, PEs were 39.4 years old (SD = 12.2), while the mean age for site leaders was 45.7 years (SD = 9.6). Most were white (72.5% for PEs and 80.8% for site leaders) and a third of the PEs and 11.5% of site leaders identified as Hispanic or Latino. The average number of years PEs had worked at their site was lower (4.6, SD 5.5) than for site leaders (11.0, SD,8.2).

**Table 1:** Baseline demographics for parent educators (provider level) and site leaders (organization level) who completed baseline surveys as part of a trial testing the effect of the HEALTH intervention.

Characteristic	Parent educators	Site leaders
Number consented	284	30
Number (%) who responded to the baseline survey	271 (95)	26 (87)
Number who completed the baseline demographic form	269^b^	26^c^
Age (years), mean (SD)	39.4 (12.2)	45.7 (9.6)
Gender, *n* (%)		
Male	1 (0.4)	0 (0.0)
Female	268 (99.6)	26 (100.0)
Race, *n* (%)		
Black or African American	27 (10.0)	3 (11.5)
White	195 (72.5)	21 (80.8)
Other^a^	11 (4.1)	0 (0)
Unknown, not reporting race	36 (13.4)	2 (7.7)
Ethnicity, *n* (%)		
Hispanic or Latino	90 (33.5)	3 (11.5)
Not Hispanic or Latino	172 (63.9)	22 (84.6)
Unknown, not reporting ethnicity	7 (2.6)	1 (3.8)
Highest grade of school completed, *n* (%)		
Less than high school/some high school/high school graduate or GED/some college, *n* (%)	42 (15.6)	0 (0)
College or university graduate/technical or vocational school	186 (69.1)	12 (46.1)
Graduate or professional school	41 (15.2)	14 (53.8)
Years working at site, mean (SD) (*n* = 265)	4.6 (5.5)	11 (8.2)
Years working in current position, mean (SD) (*n* = 265)	4.2 (5.3)	7.2 (7.4)

*n*, sample size; SD, standard deviation.

^a^ Other race includes American Indian or Alaska Native, Asian, Native Hawaiian or Other Pacific Islander, and more than one race.

^b^ The sample size (*n*) for a characteristic for parent educator is also reported if it is less than 269.

^c^ The sample size (*n*) for a characteristic item for site leader is also reported if it is less than 26.

### Reliability (Cronbach's alpha) and agreement (ICCs): CFIR context—provider and organization levels

A complete list of the scales, individual questions making up these scales, and citations for the source of the measure is provided, with the CFIR constructs they are measuring, in Table 2. Based on the alphas, most scales were Adequate to Good. Only two scales reached the level of ≥0.90 or Excellent, none were Poor, and only one was Minimal/Emerging (72). For measures at the site level, alphas for nearly all scales ranged between 0.80 and 0.89 (Good), with one scale <0.50 (Minimal/Emerging). At the PE level, two alphas were ≥0.90 (Excellent), while one had an alpha of 0.76 (Adequate). When considering agreement between PEs within PAT sites, the ICCs for all scales assessing CFIR constructs were very small (range: 0–0.2, Table 2), suggesting substantial variation in CFIR contextual factors across PEs within sites.

**Table 2:** Descriptive statistics of CFIR (the Consolidated Framework for Implementation Research) implementation context scales among parent educators (provider level) and site leaders (organization level) who completed baseline surveys as part of a trial testing the effect of the HEALTH intervention.^a^

Data level	Measurements	Standardized alpha^d^
	Individuals domain: innovation deliverers (provider level)	
PE	PE characteristics: knowledge and beliefs about HEALTH (89)	**0** **.** **76**
	1. I do not know what a Healthy Lifestyle curriculum is.^b^^,^^c^	0.81
	2. I am aware of curricula which address healthy weight.	0.64
	3. I can distinguish between different curricula which address healthy weight.	0.67
	4. I know the status of healthy weight education in my site.	0.69
	CFIR: Awareness Score: *N* = 267, Median=2.75, IQR=(2.25, 3.25), Range=(1, 4), ICC=0.19	
	1 = strongly disagree, 2 = somewhat disagree, 3 = somewhat agree, 4 = strongly agree	
PE	PE characteristics: self-efficacy (90, 91)	**0** **.** **92**
	1. Parent educators at our site understand what is required in order to implement HEALTH as prescribed.	0.88
	2. Parent educators at our site have a clear understanding of what is involved in order to fully implement HEALTH as prescribed.	0.88
	3. Our site has the expertise needed in order to implement HEALTH as prescribed.	0.90
	4. I am confident that I can implement HEALTH as prescribed at our site.	0.92
	CFIR: Self-efficacy Score: *N* = 250, Median=5.5, IQR=(4.5, 6), Range=(1, 7), ICC=0.2	
	1 = strongly disagree, 2 = disagree, 3 = somewhat disagree, 4 = neither agree or disagree, 5 = somewhat agree, 6 = agree, 7 = strongly agree	
	Inner setting domain (site/organization level)	
SL	Culture (58)	**0** **.** **81**
	1. People at all levels of our site openly talk about what is and is not working	0.79
	2. Most people at our site are willing to change how they do things in response to feedback from others	0.79
	3. It is hard to get things to change at our site	0.82
	4. I can rely on the other people at our site to do their jobs well	0.76
	5. Most of the people who work at our site seem to enjoy their work	0.78
	6. Difficult problems are solved through face-to-face discussions at our site	0.81
	7. People at our site regularly take time to reflect on how we do things	0.80
	8. After trying something new, people at our site take time to think about how it worked	0.82
	9. People at our site operate as a real team	0.76
	CFIR: culture score: *N* = 26 Mean=4.24 SD = 0.38 Range=(3.67, 5)	
	1 = Strongly disagree 2 = Disagree 3 = Neutral 4 = Agree 5 = Strongly agree	
SL	Culture: learning-centeredness (58)	**0** **.** **83**
	1. People at our site regularly take time to consider ways to improve how we do things	0.80
	2. People at our site actively seek new ways to improve how we do things	0.78
	3. Our site encourages everyone to share ideas	0.84
	4. Our site learns from its mistakes	0.79
	5. When we experience a problem at our site, we make a serious effort to figure out what is really going on	0.77
	CFIR: learning climate score: *N* = 26, median=4.4, IQR=(4, 4.6), Range=(3.6, 5)	
	1 = strongly disagree, 2 = disagree, 3 = neutral, 4 = agree, 5 = strongly agree	
SL	Culture: deliver-centeredness: Leadership engagement (58)	**0** **.** **86**
	6. Leadership at our site creates an environment where things can be accomplished	0.84
	7. Site leadership promotes an environment that is an enjoyable place to work	0.76
	8. Site leadership strongly supports site change efforts	0.77
	5. The site leadership makes sure that we have the time and space necessary to discuss changes to improve implementation of HEALTH	0.88
	CFIR: Leadership Engagement Score: *N* = 26, median=4.25, IQR=(4, 4.75), Range=(3.25, 5)	
	1 = strongly disagree, 2 = disagree, 3 = neutral, 4 = agree, 5 = strongly agree	
SL	Available resources (58)	**0** **.** **47**
	9. In general, when there is agreement that change needs to happen at our site we have the necessary support in terms of: budget or financial resources	0.50
	10. In general, when there is agreement that change needs to happen at our site we have the necessary support in terms of: training	0.44
	11. In general, when there is agreement that change needs to happen at our site we have the necessary support in terms of: staffing	0.49
	6. The following are available to make HEALTH work at our site: equipment and materials	0.43
	7. The following are available to make HEALTH work at our site: family awareness/need	0.35
	8. The following are available to make HEALTH work at our site: intervention team.	0.29
	CFIR: available resources score: *N* = 26, mean=3.6, SD = 0.42, range=(2.83, 4.5)	
	1 = strongly disagree, 2 = disagree, 3 = neutral, 4 = agree, 5 = strongly agree	
	Inner setting domain (provider level)	
PE	Mission alignment (90, 91)	**0** **.** **90**
	1. HEALTH fits well with the mission or overall goals of our site	0.87
	2. HEALTH fits well with the treatment philosophy of our site	0.87
	3. Our site is highly motivated to implement HEALTH	0.86
	4. Our site is driven to implement HEALTH	0.87
	RE-AIM: strategic fit score: *N* = 259, median=6, IQR=(5.25, 6.75), range=(1, 7) ICC=0	
	1 = strongly disagree, 2 = disagree, 3 = somewhat disagree, 4 = neither agree or disagree, 5 = somewhat agree, 6 = agree, 7 = strongly agree	
	Innovation domain (site/organization level)	
SL	Innovation relative advantage (76)	**0** **.** **88**
	1. Using HEALTH is compatible with the parent educator activities in my site	0.86
	2. I think that using HEALTH fits well with the way I like to work	0.84
	7. Using HEALTH will enhance my effectiveness on the job	0.87
	10. Using HEALTH will increase my ability to get funds for my site	0.89
	11. Using HEALTH will increase the quality of PAT programs in my site	0.86
	12. Using HEALTH will have no effect on family outcomes^b^	0.86
	14. Even if PAT National Center did not encourage the use of HEALTH, I would like to implement HEALTH in my site	0.85
	15. Overall, I find using HEALTH to be advantageous for my site	0.86
	RE-AIM: relative advantage/compatibility score: *N* = 26, mean=4.05, SD = 0.5, range=(3.13, 4.75)	
	1 = strongly disagree, 2 = disagree, 3 = neither agree nor disagree, 4 = agree, 5 = strongly agree	
SL	Innovation complexity (76)	**0** **.** **82**
	3. I believe that using HEALTH would require my site to make substantial changes to our present program^b^	0.77
	4. It will be difficult to train parent educators to implement HEALTH^b^	0.81
	5. Overall, I believe that it will be complicated to implement HEALTH^b^	0.72
	13. HEALTH requires more work than can be done with current funding^b^	0.78
	RE-AIM: complexity score: *N* = 26, mean=3.95, SD = 0.64, range=(3, 5)	
	1 = strongly disagree 2 = disagree, 3 = neither agree nor disagree, 4 = agree, 5 = strongly agree	

PE, parent educators; SL, site leader; PAT, Parents as Teachers; HEALTH, Healthy Eating and Active Living Taught at Home; IQR, interquartile range defined as the 25th and 75th percentiles; *N*, sample size; SD, standard deviation; ICC, intraclass correlation coefficient.

^a^ For each domain, standardized Cronbach's alpha is reported for each scale since the variance of variables within a scale varies widely. Within each scale, standardized alpha by deleting the item is reported for each item followed by summary statistics for the CFIR scale score.

^b^ Questions were reversed coded to calculate Cronbach's alpha and derive summary score.

^c^ Question was not reverse coded in the reference paper. However, we reversed coded based on common sense.

^d^ Bolded values are the alpha for the scale consisting of all items; unbolded values are the alpha for the scale after deleting the corresponding item.

### Reliability (Cronbach's alpha): CFIR antecedent outcomes—organization level

A complete list of the scales and individual questions making up CFIR antecedent outcomes is provided in Table 3. Two scales measuring antecedent outcomes had alphas that were Excellent (>0.9), while the implementation climate scale had a Minimal/Emerging alpha (0.64).

**Table 3:** Descriptive statistics of antecedent assessments scales among site leaders (organization level) who completed baseline surveys as part of a trial testing the effect of the HEALTH intervention.^a^

Data Level	Measurements	Standardized Alpha^b^
SL	Implementation climate (58)	**0** **.** **64**
	1. Site staff are expected to help HEALTH meet its goal (i.e., promote healthy weight).	0.63
	2. Site staff get the support they need to implement HEALTH.	0.63
	3. Site staff get recognition for implementing HEALTH to promote healthy weight.	0.48
	4. HEALTH to promote healthy weight is a top priority of our site.	0.54
	CFIR: Implementation Climate Score: *N* = 26, mean=3.86, SD = 0.47, range=(3, 5)	
	1 = strongly disagree, 2 = disagree, 3 = neutral, 4 = agree, 5 = strongly agree	
SL	Change commitment (77)	**0** **.** **91**
	1. People who work at our site are committed to implementing HEALTH	0.89
	4. People who work at our site want to implement HEALTH	0.85
	6. People who work at our site are determined to implement HEALTH	0.84
	8. People who work at our site are motivated to implement HEALTH	0.94
	CFIR: Change Commitment Score: *N* = 26, median = 4.625, IQR=(3.75, 5), range=(3, 5)	
	1 = disagree, 2 = somewhat disagree, 3 = neither agree or disagree, 4 = somewhat agree, 5 = agree	
SL	CFIR: change efficacy (77)	**0** **.** **91**
	2. People who work at our site feel confident that they can keep track of progress in implementing HEALTH	0.88
	3. People who work at our site feel confident that the organization can support people as they adjust to HEALTH	0.87
	5. People who work at our site feel confident that they can handle the challenges that might arise in implementing HEALTH	0.86
	7. People who work at our site feel confident that they can coordinate tasks so that HEALTH implementation goes smoothly	0.86
	9. People who work at our site feel confident that they can manage the politics of implementing HEALTH	0.94
	CFIR: Change efficacy score: *N* = 26, median=4.4, IQR=(3.8, 4.8), range=(3, 5)	
	1 = disagree, 2 = somewhat disagree, 3 = neither agree or disagree, 4 = somewhat agree, 5 = agree	

SL, site leader; PAT, Parents as Teachers; HEALTH, Healthy Eating and Active Living Taught at Home; IQR, interquartile range and ranges; *n*, sample size.

^a^ Standardized Cronbach's Alpha is reported for each scale since the variance of variables within a construct varies widely. Within each scale, standardized alpha by deleting the item is reported for each item followed by summary statistics for the CFIR antecedent outcomes scale score.

^b^ Bolded values are the alpha for the scale consisting of all items; unbolded values are the alpha for the scale after deleting the corresponding item.

### Reliability (Cronbach's alpha) and agreement (ICCs): RE-AIM outcomes—provider and organization levels

A complete list of the scales and individual questions measuring the RE-AIM implementation outcomes constructs is provided in Table 4. At the PE level, the two scales assessing the Adoption constructs had alphas of 0.84 (Good) and 0.90 (Excellent) and one scale measuring Implementation/Acceptability had an alpha of 0.85 (Good). However, for the other Implementation/Acceptability scale, the alpha was only 0.70 (Adequate). At the site leader level, alphas for the implementation outcomes scales ranged from 0.66 to 0.95. For the measures assessing RE-AIM outcomes, the ICCs for the scales ranged from <0.01 to 0.24. Among these, the Implementation/Acceptability measures demonstrated greater, though still low, agreement (ICCs ≥ 0.10, Table 4).

**Table 4:** Descriptive statistics of RE-AIM (Reach, Effectiveness, Adoption, Implementation, and Maintenance) implementation outcomes scales among parent educators (provider level) and site leaders (organization level) who completed baseline surveys as part of a trial testing the effect of the HEALTH intervention.^a^

Data Level	Measurements^c^	Standardized Alpha^d^
If you received training in a curriculum that was new to you, how likely would you be to adopt it if…		
	Adoption (78)	
**PE**	Appeal	**0**.**84**
	1. It was intuitively appealing?	0.81
	2. It made sense to you?	0.76
	6. It was being used by colleagues who were happy with it?	0.81
	7. You felt you had enough training to use it correctly?	0.78
	RE-AIM: Appeal Score: *N* = 266 Median=3 IQR=(2.5, 3.75) Range=(0, 4) ICC=0	
**PE**	Requirement (78)	**0**.**90**
	3. It was required by your supervisor?	0.82
	4. It was required by your site?	0.78
	5. It was required by your state?	0.96
	RE-AIM: Requirement Score: *N* = 268 Median=3 IQR=(2, 4) Range=(0, 4) ICC=0.03	
**SL**	Appeal (78)	**0**.**66**
	1. It was intuitively appealing?	0.61
	2. It made sense to you?	0.38
	6. It was being used by colleagues who were happy with it?	0.70
	7. You felt you had enough training to use it correctly?	0.61
	RE-AIM: appeal score: *N* = 26, median=3.5, IQR=(3, 3.75), range=(2.25, 4)	
**SL**	Requirement (78)	**0**.**95**
	3. It was required by your supervisor?	0.91
	4. It was required by your site?	0.93
	5. It was required by your state?	0.93
	RE-AIM: Requirement Score: *N* = 26, median=4, IQR=(2, 4), Range=(0, 4)	
	Implementation/Acceptability	
**PE**	Open (78)	**0**.**85**
	1. I like to use a new curriculum to help families.	0.80
	2. I am willing to try a new curriculum even if I have to follow a manual.	0.77
	4. I am willing to use a new and different curriculum developed by researchers.	0.81
	RE-AIM: Openness Score: *N* = 268, Median=3, IQR=(2.67, 4), Range = (1, 4), ICC=0.24	
**PE**	Divergence (78)	**0**.**70**
	3. I know better than academic researchers how to implement HEALTH for families.^b^	0.67
	5. A research based curriculum is not useful.^b^	0.58
	6. Experience working in the field is more important than using a manualized curriculum.^b^	0.72
	7. I would not use a manualized curriculum.^b^	0.58
	RE-AIM: Divergence Score: *N* = 265, median=3.25, IQR=(2.75, 3.75), range=(0, 4) ICC=0.13	

PE, parent educators; SL, site leader; IQR, interquartile range and ranges; *n*, sample size ICC, intraclass correlation coefficient.

^a^ For each domain, standardized Cronbach's alpha statistics are reported for each scale since the variance of variables within a construct varies widely. Within each scale and for each group of participants, standardized alpha statistics by deleting the item are reported for each item followed by summary statistics for the RE-AIM scale score.

^b^ Questions were reversed coded to calculate Cronbach's alpha and derive summary score.

^c^ Response items for all items: 0 = not at all, 1 = to a slight extent, 2 = to a moderate extent, 3 = to a great extent, 4 = to a very great extent.

^d^ Bolded values are the alpha for the scale consisting of all items; unbolded values are the alpha for the scale after deleting the corresponding item.

### CFIR context and RE-AIM outcomes—provider level

Table 5 presents correlations between CFIR context and RE-AIM outcomes at the parent educator level. All correlations within CFIR context were statistically significant and greater than 0.3. Within RE-AIM domains, several were statistically significantly correlated, though the correlations were generally lower than within the CFIR context. The limited within-framework relationships suggest that the RE-AIM domains may be measuring more distinct factors, while those with CFIR may have more overlap. Looking across frameworks, several correlations were statistically significant. All three CFIR context measures were statistically significantly correlated with the RE-AIM implementation outcome. CFIR knowledge and beliefs about the intervention were significantly correlated with RE-AIM outcomes of adoption and implementation. These findings may suggest relationships between contextual determinants and implementation outcomes, as depicted in Supplementary Figure 1 (Supplementary File 2). However, all correlations were low in magnitude.

**Table 5:** Correlations between measures of CFIR context constructs and RE-AIM (Reach, Effectiveness, Adoption, Implementation, and Maintenance) implementation outcomes among parent educators (provider level) who completed baseline surveys as part of a trial testing the effect of the HEALTH intervention.

Framework	Domain/Construct	CFIR context			RE-AIM outcomes			
		Knowledge and beliefs about HEALTH	Self-efficacy	Mission alignment	Adoption (appeal)	Adoption (require)	Implementation (openness)	Implementation (divergence)
CFIR Context	Knowledge and beliefs about HEALTH	1.0	**0** **.** **46**	**0** **.** **34**	0.044	**−0** **.** **19**	**0** **.** **29**	**–0** **.** **24**
	Self-efficacy		1.0	**0** **.** **51**	**0** **.** **15**	–0.056	**0** **.** **36**	–0.16
	Mission alignment			1.0	**0** **.** **13**	0.031	**0** **.** **34**	0.00
RE-AIM Outcomes	Adoption (appeal)				1.0	**0** **.** **38**	**0** **.** **39**	–0.012
	Adoption (require)					1.0	0.079	0.076
	Implementation (openness)						1.0	–0.095
	Implementation (divergence)							1.0

CFIR, Consolidated Framework for Implementation Research; RE-AIM, Reach, Effectiveness, Adoption, Implementation, and Maintenance (RE-AIM).

Bold indicates *p* < .05; light gray highlight is within-context correlations, dark gray highlighting is-within outcome correlations.

### CFIR context constructs, CFIR antecedent outcomes, and RE-AIM outcomes—organization level

At the site level (completed by one site leader at each site, Table 6), the correlations between the constructs within the CFIR framework ranged from 0.28 to 0.69, similar in magnitude to those observed at the PE level. All but three correlations were statistically significant. When exploring antecedent outcomes and RE-AIM, there were high statistically significant correlations within frameworks. As anticipated based on the CFIR outcomes addendum and Supplementary Figure 1 (Supplementary File 2), several measures of CFIR constructs (Leadership engagement, Available Resources, and Relative Advantage) were significantly associated with all three antecedent outcomes measures. Further, the Appeal measure of Adoption from the RE-AIM framework was statistically significantly correlated with all the measures of CFIR constructs, except for Innovation Complexity. However, the Require measure of Adoption was not significantly associated with any CFIR constructs. Finally, though the antecedent outcomes were associated with the measures of the CFIR constructs, the measures of the antecedent outcomes were not statistically significantly correlated with the measures of Adoption for RE-AIM. This contrasts with the relationships articulated in Supplementary Figure 1 (Supplemental File 2).

**Table 6:** Correlations between measures of CFIR context constructs, CFIR antecedent assessments, and RE-AIM (Reach, Effectiveness, Adoption, Implementation, and Maintenance) implementation outcomes among site leaders (organization level) who completed baseline surveys as part of a trial testing the effect of the HEALTH intervention.

Framework	Domain/Construct	CFIR context						Antecedent assessments			RE: AIM outcomes	
		Culture	Learning climate	Leadership engagement	Available resources	Relative advantage	Innovation complexity	Implementation climate	Change commitment	Change efficacy	Adoption (appeal)	Adoption (require)
CFIR Context	Culture	1	**0** **.** **51**	**0** **.** **50**	0**.**28	**0** **.** **41**	**0** **.** **50**	0**.**19	0**.**36	0**.**36	**0** **.** **54**	0**.**17
	learning Climate		1	**0** **.** **69**	**0** **.** **46**	**0** **.** **47**	0**.**36	0**.**30	0**.**38	0**.**36	**0** **.** **61**	0**.**38
	leadership Engagement			1	**0** **.** **57**	**0** **.** **54**	**0** **.** **49**	**0** **.** **45**	**0** **.** **44**	**0** **.** **43**	**0** **.** **58**	0**.**27
	Available resources				1	**0** **.** **49**	0**.**37	**0** **.** **55**	**0** **.** **55**	**0** **.** **54**	0**.**10	-0**.**27
	Relative advantage					1	**0** **.** **50**	**0** **.** **56**	**0** **.** **67**	**0** **.** **63**	**0** **.** **46**	-0**.**12
	Innovation Complexity						1	0**.**017	0**.**38	**0** **.** **51**	0**.**36	-0**.**013
Antecedent Assessments	implementation Climate							1	**0** **.** **66**	**0** **.** **55**	0**.**29	0**.**074
	change Commitment								1	**0** **.** **94**	0**.**35	0**.**015
	Change efficacy									1	0**.**26	-0**.**022
RE: AIM Outcomes	Adoption (appeal)										1	**0** **.** **67**
	Adoption (require)											1

CFIR, Consolidated Framework for Implementation Research; RE-AIM, Reach, Effectiveness, Adoption, Implementation, and Maintenance (RE-AIM).

Bold indicates *p* < .05; light gray highlight is within-context correlations, blue highlight is within-antecedent assessment correlation, dark gray highlight is within-outcome correlations.

## Discussion

This study applied implementation science frameworks (i.e., CFIR and RE-AIM), described survey measures used to assess the framework constructs, and explored correlations within and across the frameworks in a community-based home-visiting organization. Surveys to assess constructs from CFIR context, CFIR antecedent outcomes, and RE-AIM implementation outcomes include seven scales at the PE level and 11 scales at the site level. Most scales had alphas that demonstrated Good to Excellent internal consistency reliability (72), while low ICCs suggested limited agreement among PEs at a site (relative to between sites). At the PE level, there were consistently positive, statistically significant correlations within the surveys assessing the CFIR framework constructs, while correlations with most of the measures assessing RE-AIM domains (except for the openness measure of implementation) were low, not statistically significant, and inconsistent in direction. This was similar at the site level, which also found positive, statistically significant relationships between measures of CIFR context constructs and measures of CFIR antecedent outcomes.

These findings underscore the value of implementation science in enhancing the rigor of community-based healthy eating and activity research. Implementation science measures can help capture the complexities of implementation in settings often designed to address social determinants of health (e.g., education, employment), rather than health promotion content (10, 15, 75). Compared with prior studies, the internal consistency reliability of the CFIR context (58, 76), CFIR antecedent outcomes (58, 77), and RE-AIM outcomes (78–81) measures in the current sample was quite similar, with most scales achieving at least Good reliability (Cronbach's alpha ≥ 0.8). One notable exception was the low alpha for the Available Resources measure, and researchers may consider alternative measures for future work. These findings may be due to the items making up this scale. In the set of items, there are two stems “In general, when there is agreement that change needs to happen at our site we have the necessary support in terms of:” and “The following are available to make HEALTH work at our site:”. Such wording may lead participants to provide similar answers across items (82).

The low ICCs indicating limited agreement among PEs have several implications. First, limited agreement within sites suggests that contextual constructs (e.g., perceptions of the healthy eating and activity intervention) and implementation outcomes may reflect individual provider perspectives. This indicates that these constructs need to be measured at the provider level, rather than aggregating within a site. Further, reporting agreement among community-based providers within sites can inform the design of clustered studies (20), including design decisions about whether to assess implementation science constructs at the cluster (site) level. To our knowledge, no other studies have reported agreement among similar types of providers, limiting comparisons of the current findings.

While the current study found associations among measures assessing constructs within CFIR and between the measures of CFIR context and the measures of CFIR antecedent outcomes [which is suggested by the CFIR Outcomes Addendum (35)], it did not identify consistent, statistically significant associations between the measures of CFIR context and CFIR antecedent assessments and measures of the RE-AIM domains. The one exception was the openness measure of implementation (an RE-AIM domain) and each of the measures of CFIR contextual constructs, at the PE level. These findings contrast with the hypothesized relationships in Supplementary Figure 1 (Supplementary File 2) and highlight a limitation of relying on frameworks, which do not hypothesize relationships between constructs and domains (26). This is also somewhat different from the pattern that has been found in previous literature. For example, Wilcox et al. used the CFIR and RE-AIM frameworks to understand dissemination and implementation of a nutrition and physical activity intervention in churches in South Carolina. They found that constructs from the CFIR (e.g., relative advantage, relative priority, self-efficacy of implementers) predicted intervention sustainment (33, 51, 53, 83, 84). Similarly, work by Swindle et al. emphasized the importance of constructs such as organizational capacity and environmental support from the Dynamic Sustainability Framework (an implementation science framework) for sustaining adherence to nutrition and physical activity promotion program designed within early care and education settings (62, 85, 86). While the associations between CFIR context and implementation outcomes in the present study are associative and not predictive, these results contribute to the growing body of research suggesting that assessing constructs from implementation science frameworks across community-based settings can help build a generalizable understanding of implementation context and outcomes for healthy eating and activity interventions.

### Limitations

This study has limitations related to sample size and representation, specific measures tested for CFIR/RE-AIM constructs, reliance on self-report, limited validity testing, no correction for multiple comparisons, and the cross-sectional study design. This study is limited by the various *sample sizes* of PEs within sites and by the sample size of <30 for the site-level measures. In addition, while the sample comprised providers from different racial, ethnic, and education backgrounds, most participants were female, white, non-Hispanic, and more educated, suggesting the need to validate these measures in underrepresented groups. Further, while *the measures* were based on previous studies, they were adapted for the current study and were initially mapped to the constructs in the original version of CFIR. They were then reassessed based on the updated CFIR. These mappings were also determined for the current study, as there is limited guidance on specific measures to assess the constructs in implementation science theories, models, and frameworks, particularly in community settings. The study assessed a limited set of properties regarding the measures presented. For example, the constructs were assessed using *self-report measures*, which reflect respondents’ perceptions. Criterion *validity* requires evidence of relationships between the measure and another measure assessing a different context (72). However, due to the lack of “gold standard” measures with which to assess these constructs, criterion validity is difficult to assess. Other types of validity assessment are also somewhat limited by the current state of the implementation science field. For example, construct validity (which describes how well a measure assesses the theoretical concept it is supposed to measure) is also challenging to assess, as the measures are assessing framework constructs, but the theory of how the constructs are related remains limited. Further work on theory building, perhaps using the constructs as measured by the scales described in the current paper, may help address this gap, but is beyond the scope of the current manuscript. Given the limited theoretical guidance, the study aims were to generate a hypothesis, rather than to confirm one. Therefore, the current, exploratory analysis examined numerous correlations and did not adjust the *p*-values for *multiple comparisons*. Finally, since this was a *cross-sectional study*, temporality and causal relationships between CFIR contextual determinants and CFIR and RE-AIM outcomes could not be tested. Longitudinal follow-up from this and other studies using these frameworks and measures may provide more insight into the relationships between implementation science determinants and outcomes in community settings.

### Strengths

The study is strengthened by the large sample size of PEs, the number of participating sites, and the geographic diversity of the participants. Furthermore, the study includes measurement at multiple levels (i.e., provider and organization). Importantly, the study provides psychometric properties of theoretically informed measures in under-studied community settings, which are essential for meeting people where they are and building generalizable implementation science. Quantitative data from these measures can complement existing qualitative research (44, 84, 87). As much implementation work has focused primarily on qualitative approaches, these quantitative assessments allow for mixed-methods studies (38, 88), which can develop a rich understanding of the implementation of healthy eating and activity interventions outside clinical settings.

## Conclusions

The present study operationalizes frameworks from implementation science (CFIR and RE-AIM) within a community-based home-visiting program. This study advances implementation science by providing psychometric evidence for measuring implementation in community settings as well as by testing and bridging implementation science determinants and outcomes frameworks. Researchers can use these findings to select measures for future studies and maximize rigor by operationalizing implementation science frameworks through measurement of their constructs (57).

## Data Availability

The datasets used/analyzed during the current study are available from the corresponding author on reasonable request. Requests to access the datasets should be directed to Rachel Tabak, rtabak@wustl.edu.
