# Supplementary material for: Application of implementation science frameworks to a community-based healthy eating and activity intervention: a cross-sectional analysis
Source: Front Health Serv. 2026 Feb 18;6:1637060. doi: 10.3389/frhs.2026.1637060 (PMC12958059; doi:10.3389/frhs.2026.1637060)
Supplement: Supplementary file 1 [file Table1.docx]

**Supplemental Table 1. Names and definitions of constructs from CFIR (the Consolidated Framework for Implementation Research) and RE-AIM (Reach, Effectiveness, Adoption, Implementation, and Maintenance)**

| **Construct name** | **Level** | **Definition** |
| --- | --- | --- |
| **CFIR implementation determinant scales** | | |
| **Individuals Domain: Innovation Deliverers (provider level)** | | |
| PE Characteristics: Knowledge and Beliefs About HEALTH (Steckler et al., 1992) | PE | PE’s attitudes toward and value placed on HEALTH and familiarity with facts, truths, and principles related to HEALTH |
| PE Characteristics: Self-Efficacy (Massatti et al., 2008; Panzano et al., 2012) | PE | PE belief in their own capabilities to achieve implementation goals |
| **Inner Setting Domain (site/organization level)** | | |
| Culture (Fernandez et al., 2018) | SL | Norms, values, and basic assumptions of a PAT site |
| Culture: Learning-Centeredness (Fernandez et al., 2018) | SL |  |
| Culture: Deliver-Centeredness: Leadership Engagement (Fernandez et al., 2018) | SL |  |
| Available Resources (Fernandez et al., 2018) | SL | Resources are available to implement and deliver HEALTH |
| **Inner Setting Domain (provider level)** | | |
| Mission Alignment (Massatti et al., 2008; Panzano et al., 2012) | PE | Perceived fit, relevance, and compatibility of HEALTH for PAT and parent educators; and perceived fit of HEALTH to address weight |
| **Innovation Domain (site/organization level)** | | |
| Innovation Relative Advantage (Pankratz et al., 2002) | SL | Parent educators’ perception of the advantage of implementing HEALTH versus usual care |
| Innovation Complexity (Pankratz et al., 2002) | SL | Perceived difficulty of implementation, reflected by duration, scope, radicalness, disruptiveness, centrality, intricacy, and number of steps required |
| **Antecedent assessments scales at the site/organizational level** | | |
| Implementation Climate (Fernandez et al., 2018) | SL | Absorptive capacity for change, shared receptivity of involved individuals to an intervention, and the extent to which use of the intervention will be rewarded, supported, and expected in the PAT site |
| Readiness for Implementation: Change Commitment (Shea et al., 2014) | SL | Tangible, immediate indicators of organizational commitment to its decision to implement HEALTH |
| Readiness for Implementation: CFIR: Change Efficacy (Shea et al., 2014) | SL |  |
| **RE-AIM Implementation outcomes scales** | | |
| **Adoption** (Aarons, 2004) |  | Intention, initial decision, or action to try or employ HEALTH; ‘‘uptake’’ |
| Appeal | PE/SL | HEALTH’s intuitive Appeal |
| Requirement (Aarons, 2004) | PE/SL | likelihood of adopting HEALTH given Requirements to do so |
| **Implementation/Acceptability** | | |
| Open (Aarons, 2004) | PE | Openness to new practices |
| Divergence (Aarons, 2004) | PE | perceived Divergence of usual practice with research-based/academically developed interventions |

Abbreviations: SL, site leader; PAT, Parents as Teachers; HEALTH, Healthy Eating and Active Living Taught at Home.
