# Supplementary figures and images for: Application of implementation science frameworks to a community-based healthy eating and activity intervention: a cross-sectional analysis
Source: Front Health Serv. 2026 Feb 18;6:1637060. doi: 10.3389/frhs.2026.1637060 (PMC12958059; doi:10.3389/frhs.2026.1637060)

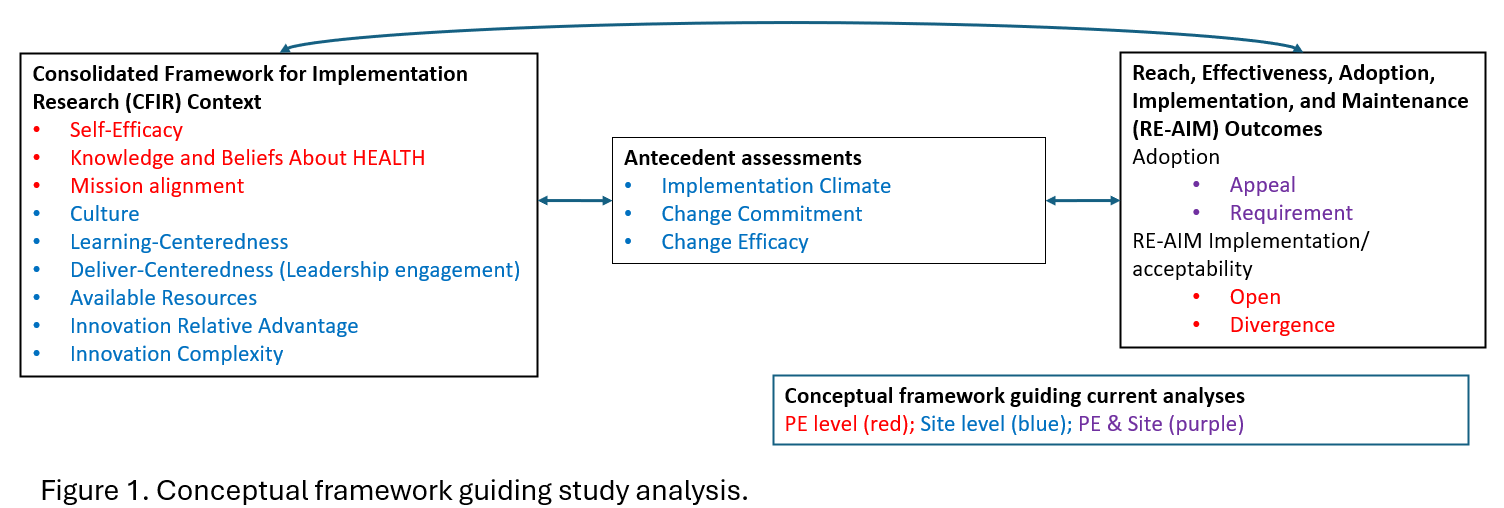

Supplement: Supplementary file 2 [file Image1.png]
